# Reply to: Response and remission in asthma with tezepelumab: overlapping concepts informing on type-2 inflammatory-dependent treatment effects

**DOI:** 10.1183/13993003.02434-2024

**Published:** 2024-02-13

**Authors:** Neil Martin, Michael E. Wechsler, Christopher E. Brightling

**Affiliations:** 1 Institute for Lung Health, National Institute for Health and Care Research, Leicester Biomedical Research Centre, University of Leicester, Leicester, UK; 2 Respiratory and Immunology, BioPharmaceuticals Medical, AstraZeneca, Cambridge, UK; 3 National Jewish Health, Denver, CO, USA

## Abstract

We thank S. Mailhot-Larouche and co-workers for their interest in our recent publication on clinical response and clinical remission with tezepelumab treatment, reporting results over 2 years from patients with severe, uncontrolled asthma enrolled in the NAVIGATOR and DESTINATION clinical trials [1].

## Shareable abstract

**Achieving on-treatment clinical remission with tezepelumab was associated with high baseline inflammatory biomarkers, but those achieving remission were observed to have less severe disease at baseline than those who achieved complete clinical response** https://bit.ly/3P0C52q

*Reply to S. Mailhot-Larouche and co-workers:*

We thank S. Mailhot-Larouche and co-workers for their interest in our recent publication on clinical response and clinical remission with tezepelumab treatment, reporting results over 2 years from patients with severe, uncontrolled asthma enrolled in the NAVIGATOR and DESTINATION clinical trials [1].

We descriptively compared the baseline characteristics of patients achieving complete clinical response at week 52 and those achieving clinical remission at week 52 to identify potential differences that may be associated with an increased likelihood of achieving complete clinical response or clinical remission with tezepelumab. We also assessed whether any baseline characteristics aligned with predictors of clinical response or clinical remission that have been identified in other studies [2–4]. It has been established that remission is more likely to be achieved in patients who have less severe damage before commencing biologic treatment [2, 4, 5]. First, we evaluated the baseline characteristics associated with achieving clinical response and clinical remission separately. We found that a greater proportion of complete clinical responders in the tezepelumab *versus* placebo group were receiving maintenance oral corticosteroids at baseline, had more than two exacerbations in the 12 months before the study and had higher baseline blood eosinophil counts (BECs) and baseline fractional exhaled nitric oxide (*F*_ENO_) levels, *i.e.* patients with more severe, uncontrolled disease and elevated inflammation at baseline. For on-treatment clinical remission, S. Mailhot-Larouche and co-workers are correct that we observed that achieving remission was associated with higher BECs and *F*_ENO_ levels at baseline. Finally, when comparing patients achieving clinical remission and complete clinical response at week 52, we found that those achieving remission were more likely to have less severe disease and lower inflammatory biomarker levels at baseline (*e.g.* better-preserved lung function, fewer exacerbations in the 12 months before the study and lower BECs and *F*_ENO_ levels).

We also note that BECs and *F*_ENO_ levels were reduced with tezepelumab treatment *versus* placebo from baseline to week 104, irrespective of whether the patients achieved on-treatment clinical remission at week 104. This suggests that achieving low levels of type 2 inflammation (*i.e.* biological remission) after treatment does not necessarily predict clinical remission. However, it remains to be fully understood how changes in biomarker levels following thymic stromal lymphopoietin blockade with tezepelumab relate to clinical response and remission outcomes, and whether biomarker reductions are required for clinical remission.

We agree with S. Mailhot-Larouche and co-workers that type 2 inflammatory biomarkers can be used to predict both clinical response and clinical remission, and that risk prediction modelling is required in asthma to allow us to treat patients earlier in the disease development, before the disease becomes severe and less reversible. We also agree that other factors such as lung damage, obesity and duration of severe disease can affect a patient's ability to achieve remission.

In our analysis, no statistical tests were performed on the baseline characteristics of those who achieved complete clinical response *versus* clinical remission at week 52, because the baseline characteristics are provided as descriptive statistics only. Additionally, achieving complete clinical response *versus* clinical remission at week 52 are not mutually exclusive because we evaluated all patients from the NAVIGATOR population at week 52 and some patients will have achieved both the complete clinical response and clinical remission criteria.

In conclusion, we agree with S. Mailhot-Larouche and co-workers that more investigatory work is needed to understand the role of biomarkers and other factors at baseline in predicting response and remission outcomes with tezepelumab treatment, as well as the impact of earlier intervention with tezepelumab on preventing long-term damage resulting from prolonged inflammation. The impact of earlier intervention with tezepelumab is being further explored in ongoing studies such as the ARRIVAL study (NCT06473779) [6], which will examine the effect of tezepelumab on clinical remission in patients with a shorter duration of asthma than presented in the NAVIGATOR and DESTINATION studies. This will grant valuable insights into earlier biologic intervention, particularly for those with severe disease, and provide more information on potential predictors of remission.

## Shareable PDF

10.1183/13993003.02434-2024.Shareable1 This PDF extract can be shared freely online.
Shareable PDF ERJ-02434-2024.Shareable

## References

[1] Wechsler ME, Brusselle G, Virchow JC, et al. Clinical response and on-treatment clinical remission with tezepelumab in a broad population of patients with severe, uncontrolled asthma: results over 2 years from the NAVIGATOR and DESTINATION studies. Eur Respir J 2024; 64: 2400316. doi:10.1183/13993003.00316-2024 39326921 PMC11618813

[2] Hansen S, Søndergaard MB, von Bülow A, et al. Clinical response and remission in patients with severe asthma treated with biologic therapies. Chest 2024; 165: 253–266. doi:10.1016/j.chest.2023.10.046 37925144

[3] Scelo G, Tran TN, Le TT, et al. Exploring definitions and predictors of response to biologics for severe asthma. J Allergy Clin Immunol Pract 2024; 12: 2347–2361. doi:10.1016/j.jaip.2024.05.016 38768896

[4] Perez-de-Llano L, Scelo G, Tran TN, et al. Exploring definitions and predictors of severe asthma clinical remission after biologic treatment in adults. Am J Respir Crit Care Med 2024; 210: 869–880. doi:10.1164/rccm.202311-2192OC 38701495 PMC11506911

[5] Shackleford A, Heaney LG, Redmond C, et al. Clinical remission attainment, definitions, and correlates among patients with severe asthma treated with biologics: a systematic review and meta-analysis. Lancet Respir Med 2025; 13: 23–34. doi:10.1016/S2213-2600(24)00293-5 39549709

[6] ClinicalTrials.gov. Open-label study to assess reduction of background asthma medication while sustaining asthma control and clinical remission with tezepelumab in patients 12–80yrs with severe asthma. (ARRIVAL). Date last accessed: 31 October 2024. Date last updated: 17 December 2024. https://clinicaltrials.gov/study/NCT06473779?rank=1

