# Supplementary material for: Reply to: Response and remission in asthma with tezepelumab: overlapping concepts informing on type-2 inflammatory-dependent treatment effects
Source: Eur Respir J. 2024 Feb 13;65(2):2402434. doi: 10.1183/13993003.02434-2024 (PMC11822240; doi:10.1183/13993003.02434-2024)
Supplement: Supplementary file 1 [file ERJ-02434-2024.Shareable.pdf]

SHAREABLE PDF

Reply to: Response and remission in asthma with tezepelumab: overlapping concepts informing on type-2 inflammatory-dependent treatment effects

Neil Martin[1,2], Michael E. Wechsler[3] and Christopher E. Brightling[1] on behalf of all authors of "Clinical response and on-treatment clinical remission with tezepelumab in a broad population of patients with severe, uncontrolled asthma: results over 2 years from the NAVIGATOR and DESTINATION studies"

[1]Institute for Lung Health, National Institute for Health and Care Research, Leicester Biomedical Research Centre, University of Leicester, Leicester, UK. [2]Respiratory and Immunology, BioPharmaceuticals Medical, AstraZeneca, Cambridge, UK. [3]National Jewish Health, Denver, CO, USA.

Corresponding author: Neil Martin (neil.martin2@astrazeneca.com)

Check for updates

Shareable abstract (@ERSpublications)
Achieving on-treatment clinical remission with tezepelumab was associated with high baseline inflammatory biomarkers, but those achieving remission were observed to have less severe disease at baseline than those who achieved complete clinical response https://bit.ly/3P0C52q

Cite this article as: Martin N, Wechsler ME, Brightling CE. Reply to: Response and remission in asthma with tezepelumab: overlapping concepts informing on type-2 inflammatory-dependent treatment effects. *Eur Respir J* 2025; 65: 2402434 [DOI: 10.1183/13993003.02434-2024].

This PDF extract can be shared freely online.

Copyright ©The authors 2025.

This version is distributed under the terms of the Creative Commons Attribution Non-Commercial Licence 4.0. For commercial reproduction rights and permissions contact permissions@ersnet.org

Received: 10 Dec 2024
Accepted: 11 Dec 2024

Reply to S. Mailhot-Larouche and co-workers:

We thank S. Mailhot-Larouche and co-workers for their interest in our recent publication on clinical response and clinical remission with tezepelumab treatment, reporting results over 2 years from patients with severe, uncontrolled asthma enrolled in the NAVIGATOR and DESTINATION clinical trials [1].
